# Lunacy in the Central Provinces of India

**Published:** 1899-04-29

**Authors:** 


### LUNACY IN THE CENTRAL PROVINCES OF INDIA.

According to the annual report of the Lunatic Asylums in the Central Provinces of India, the past four years have been marked by a steady increase in the percentage of cases cured. It should, however, be stated that this increase is partly due to the new system by which criminal lunatics upon recovery are transferred to a central gaol, there to await their trial in the ordinary way. These cases, as far as the asylums are concerned, are rightly entered in the reports as "discharged cured." Two asylums only are at present found necessary in the Central Provinces of India—one at Nagpur, which at the close of 1897 contained 205 patients; the other at Jubbulpore, with 177 inmates. As to the causes which are registered as having produced the loss of mental balance, in 175 cases they are unknown, but epilepsy and sunstroke produced 26, fever 12, and privation 6. Opium eating resulted in lunacy in four cases; spirit drinking in two. But by far the most startling is the statement that ganja smoking, it is believed, has sent more victims to the asylums than any other known cause. In one instance a youth of twenty-two confessed that he had frequented the company of Sadhus, or religious mendicants, and had taken bhang and smoked ganja in their company. This, added to religious excitement, had made him a mental wreck. Another man stated that he had been in the habit of taking ganja, bhang, and wine for years, while a third had been pursuing the same course for months only, but in every instance the effects had been equally disastrous. When first admitted these patients refused food, had to be fed by force, would wear no clothes, and could seldom be persuaded to speak. The depression was extreme, and the weakness distressing. The majority of those who came into the asylum during 1897 were found, as in previous years, to be between the ages of 20 and 40, and the labouring classes contributed the largest number of cases; cultivators, beggars, servants, and students came next upon the list. Amongst the recoveries there was one very interesting example. The man, who had been a servant for a few days to a notorious poisoner (since imprisoned), was found in some oneelse's house, having no idea how he got there, and as his brain seemed clouded he was sent to the asylum. It subsequently transpired that his master gave him some sweets to eat. He became unconscious, and knew nothing of his subsequent actions. After the use of the stomach pump he began to recover rapidly, and was soon discharged perfectly sane. Evidently the man's master had used him as a convenient victim on whom to test the effect of some new drug.

